# Morgan County Medical Society

**Published:** 1867-11

**Authors:** C. T. Wilbur

**Affiliations:** Secretary


THE SOCIETY met at the Court House, in Jacksonville, on Thursday, September 12, at 2 o'clock P.M.

The proceedings of the last meeting, as printed in the *Jacksonville Journal*, were read and approved.

Dr. Prince reported a case of epithelioma. The patient had been afflicted with occasional attacks of colic for a year, and during his last sickness was confined to his bed several months, suffering severe pain, the disease finally resulting in death. A *post mortem* examination revealed the fact that the ascending and right half of the transverse colon were very much distended, and the transverse colon was constricted at the median line by an induration of the natural tissue at the base of an epithelial tumor growing upon the interior of the intestine.

Dr. Long remarked that the patient's mother and one brother had died of obscure abdominal affections, and suggested the probability of a family predisposition to the same difficulty.

Dr. Edgar said that the patient had called his attention, within a year or two, to a tumor on the crown of the head, as large as a hickory nut—and several years ago, a lupus had been removed from his face. He thought a cancerous diathesis was clearly indicated in this case.

Owing to the intense pain which was observed, and to the absence of any signs of a tumor, the difficulty had been diagnosed as of a neuralgic character at first, and treated with quinine and iron, which really seemed to benefit the patient for a time.

Dr. Edgar thought that we should not be too easily led to jump to the conclusion of a nervous difficulty when the symptoms seem to indicate a grave organic derangement. The doctor also dwelt upon the importance of keeping a careful record of each case a physician might have—recording faithfully and accurately the various symptoms of obscure diseases, that friends and the profession might be benefited thereby.

The regularly appointed essayists, Drs. Edgar and Fisher, being unprepared, they were allowed still further time, and will be expected to furnish papers for the next meeting.

Dr. Edgar then introduced the subject of tracheotomy in croup, by relating an incident of three German physicians, who, after this operation, attempted to remove a clot of blood from the trachea by applying the lips to the opening, and who contracted the disease and died a short time after.

The President, Dr. Henry Jones, thought the disease must have been diphtheritic croup, as he had never heard or read of any evidence of the contagiousness of croup.

Dr. Prince remarked that he had performed six operations of tracheotomy for croup in children. Three were benefited temporarily, but died by the development of a similar disease in the chest. Had never operated in a case where the contagious or spreading element did not exist. If we could limit the spread of the inflammation, the operation would be successful, but the power or ability to limit this spreading element would also obviate the necessity for an operation. He thought that the best means of limiting this spreading would be an application of a strong solution of nitrate of silver to the parts by the atomizing tube.

Dr. Craig remarked that he had used tinct. muriate of iron diluted, in the form of spray.

Dr. Bibb had used a solution of nitrate of silver, in spray, with great relief in one case of croup.

Dr. DeLeuw recommended tincture of iron as an application.

Dr. Long was accustomed to using nitrate of silver.

Dr. H. K. Jones considered tracheotomy merely a mechanical means of affording relief as a last resort. Was in the habit of using in croup, calomel, tartar emetic, and ipecac, as remedies. In diphtheria, I rely upon the sesquichloride of iron. Rarely make local applications. When a pathological condition of the system exists, treat this condition and your local symptoms disappear. Consider that the severity of local disease more often depends upon some depravity of the system.

Dr. Edgar thought but little danger necessarily resulted from the operation itself, as very severe injuries were suffered in this region without producing death. He knew of a case, where not only the trachea was opened transversely, but the œsophagus also, to that extent that food passed out, and yet the patient recovered. Regarded local applications as often very beneficial. Erysipelas was often treated successfully by local means alone, without resorting to constitutional remedies.

Dr. Fisher believed in local applications in croup—though he had not used it, he looked upon the atomizing tube as a very sensible way of applying local remedies in this formidable disease. He considered diphtheria a constitutional disease, and relied upon quinine and chlorate of potash as remedies in its treatment.

Dr. Wilbur seemed to think that it was very difficult to draw the line between croup and diphtheria.

Dr. Henry Jones thought that simple croup was not contagious. Did not see any difficulty in distinguishing croup from diphtheria, as in the latter disease the throat symptoms preceded the anginous affections. Remedies should be adapted to both local and constitutional states of the system. In great emergencies, the doctor regarded the operation of tracheotomy as certainly proper, as the physician gets time for action. Croup is, originally, a local affection. I regard local applications in idiopathic erysipelas as sometimes beneficial. After the operation of tracheotomy, the treatment should aim to prevent the spread of the disease. In the sore throat of scarlet fever, by a sort of contagion, sometimes whole families will suffer from throat difficulties.

Dr. H. K. Jones did not question the expediency of tracheotomy as a means of relief. Though it sometimes gave temporary relief, the disease usually progressed and terminated fatally. Thought the best remedies were those which attacked the constitutional condition of the system which this progression proceeded from.

At 5 o'clock P.M., adjourned, to meet the second Thursday in October.

C. T. WILBUR, M.D., *Secretary.*

